# Who actualizes postpartum contraceptive intentions? A trajectory cluster analysis

**DOI:** 10.1186/s12978-024-01899-7

**Published:** 2024-11-21

**Authors:** Michelle L. O’Brien, Aasli Abdi Nur

**Affiliations:** 1 Institute for Disease Modeling, Bill & Melinda Gates Foundation, Seattle, USA; 2 Department of Sociology, Leverhulme Centre for Demographic Science and Nuffield College, University of Oxford, Oxford, UK

## Abstract

Contraceptive intention is an important woman-centered indicator for family planning. Yet, few studies have examined the determinants of women or couples *actualizing* their contraceptive intentions. We leverage panel data from the Performance Monitoring for Action (PMA) survey in Ethiopia to examine these dynamics among a pregnancy cohort, over the first year postpartum. Using cluster analysis on intent-to-use trajectories, we find distinct patterns across wealth categories, education levels, and regions. Additionally, we find that receiving family planning counseling in both antenatal and postnatal care visits led to a higher likelihood of *intending* to use. However, counseling did not increase the odds of *actualization*. We argue that examining actualization through model-based approaches like cluster analysis generates better insight into woman-centered contraceptive demand and provides stronger evidence for strengthening postpartum family planning interventions, than quantifying contraceptive use alone. Modeling postpartum actualization trajectories can shed light on the barriers to women’s and couple’s reproductive autonomy and inform future investments in both upstream development of better contraceptive methods and downstream implementation.

**Supplementary Information:**

The online version contains supplementary material available at 10.1186/s12978-024-01899-7.

## Introduction

Researchers and policymakers are increasingly recognizing the importance of women’s and couples' intentions to use contraception for understanding and meeting their family planning needs [8]. Compared to indicators such as desired fertility or unmet need, relatively few studies on contraceptive intentions and their actualization have been undertaken (e.g. [2, 5, 12, 19, 31, 33, 38]). Unlike unmet need, which is a composite measure that is supposed to approximate demand, the contraceptive intentions indicator reflects women’s expressed intentions at the individual level to understand future use. In addition to providing a more person-centered measure, contraceptive intentions have also been shown to be more predictive of future use than unmet need [34]. Despite this emerging interest in intentions, studies such as Sarnak et al. [34] designed to investigate the *actualization* of those individual-level intentions are rare. Even less focus has gone to identifying *who* actualizes their intentions and *how* they do so.

Understanding the dynamics of actualizing contraceptive intentions is critical to addressing barriers preventing women’s contraceptive access and informed choice, especially during the postpartum period. The postpartum period, also known as the “fourth trimester” [42, 46] is a critical point of intervention to improve maternal and newborn health outcomes, detect any postpartum complications, promote maternal mental health, fulfill women’s demand for contraception, and ensure optimal care for the newborn [6, 46]. Despite its importance in improving outcomes, the postpartum period remains the most neglected period in terms of the provision of quality health care when compared to pregnancy and childbirth [42, 44, 46].

Women’s use of maternal health services during and after pregnancy has been linked to improved maternal and child health outcomes, including decreased maternal and infant mortality rates [10, 25, 37]. Still, fewer women receive care from a skilled provider when compared to the rates observed during pregnancy and childbirth [45, 46]. Women’s increased contact with the healthcare system during pregnancy thus presents an opportunity to mitigate the risk of adverse events by continuing to monitor their health after childbirth. By emphasizing postnatal care alongside antenatal care, healthcare providers can comprehensively support the overall health and successful transition of women into motherhood, fostering positive outcomes for both mother and child.

The first year postpartum is also a crucial window to provide supportive care for women, including information about and access to safe and effective family planning methods, including lactational amenorrhea (LAM), effective non-hormonal methods, and pharmaceutical contraception that can be used during this period [46]. Short birth intervals resulting from unintended pregnancies too soon during the postpartum period are associated with a wide range of adverse outcomes for maternal and child health [11, 14, 35], particularly in low-income settings [29].

In this research, we examine contraceptive dynamics in this critical postpartum period. We focus on *contraceptive actualization trajectories* during the first year postpartum by leveraging panel data from a pregnancy cohort from the Performance Monitoring in Action (PMA) data collection in Ethiopia. Using cluster analysis on longitudinal trajectories, we examine women’s postpartum contraceptive intentions and subsequent use. We further examine who is able to actualize their plans. We discuss potential mechanisms that act as enablers or barriers to women’s contraceptive actualization and extend on previous actualization research by leveraging a model-based approach to define a range of potential intent-to-action trajectories. This analysis provides deeper insight into women’s actual needs and contraceptive demand postpartum.

## Context

In Ethiopia, almost half of all pregnancies occur within 24 months of the previous birth for primiparous women [3, 41]. The WHO and Ethiopia’s national family planning guidelines recommend spacing pregnancies at least 24 months apart to minimize potentially adverse effects on perinatal, neonatal, and child health outcomes [32]. Despite its importance during the postpartum period, healthcare managers, providers, and users often give less attention to family planning in the first year after childbirth [4, 32], leaving many women unaware of their risk of unintended pregnancy during this period.

Women in Ethiopia who state an intent to use contraception during the postpartum period are likely to have previous knowledge and/or experience of contraception [1, 18, 21, 39, 40, 43]. Still, they may underestimate the risk of pregnancy during this period before the return of menstruation. Following delivery, women often experience a return to fertility before starting a new method [7]. In Ethiopia, low uptake of postpartum family planning (PPFP) has been associated with a perceived lower risk of pregnancy among women who are breastfeeding and amenorrheic [32, 41]. Postpartum women also cite fear of side effects, infrequent sexual activity, and opposition from their partners as their main reasons for contraceptive non-use [13, 16, 21].

The Ethiopian Ministry of Health (MOH) has thus recommended that postpartum family planning be fully integrated into maternal health services, so that “every new mother will leave the clinic having made an informed choice about family planning,” [28]:28). An important component of the guidelines to integrate PPFP into maternal health services is quality counseling, which does seem to impact contraceptive uptake, even though rates of counseling remain low in Ethiopia [30]. The MOH advises healthcare providers to engage in discussions with expectant mothers during antenatal care visits on PPFP, including contraceptive counseling. Improving women’s access to contraception at health facilities directly after childbirth is critical to the MOH strategy for improving PPFP care, however, the vast majority of women in Ethiopia still give birth at home [20]. This gap in service delivery means that, despite the increased investment, many women may not have access to the integrated PPFP services that reduce their risk of unplanned or shortly spaced pregnancies.

Given the importance of postpartum family planning in Ethiopia, we use this case study to expand on previous work examining contraceptive intentions. In our work, we focus on women’s ability to actualize their stated intention to use. This focus allows us to identify important patterns that are associated with women’s agency and resources to enact their postpartum plans.

## Analytical approach

We use data from the longitudinal Performance Monitoring for Action (PMA) survey in Ethiopia. These data capture the first year postpartum of a pregnancy cohort (Cohort 1), with contraceptive intentions and use collected at baseline (pregnancy), 6-week, 6-month, and 1-year postpartum follow-up, for a total of four waves of data. Baseline data were collected in fall 2019 in Addis Ababa, Afar, Amhara, Oromiya, Southern Nations Nationalities and Peoples’ Region (SNNP), and Tigray^1^ with a total of 2,868 women surveyed at the baseline and 2,094 remaining by the one-year follow up. We report descriptive statistics of this sample in Table 1.

**Table 1:** Descriptive statistics of the PMA Ethiopia pregnancy cohort^a^

	Baseline	6-week follow up	6-month follow up	1-year follow up
Sample size (unweighted)	2,872	2,582	2,698	2,694
Sample size (weighted)	2,868	2,392	2,418	2,095
# maternal deaths between waves	N/A	2	2	1
Final sample size (weighted, removing deaths)	2,868	2,390	2,416	2,094
Age				
Range	15–48			
25th percentile	22			
Median	27			
75th percentile	31			
Regional distribution				
Tigray	200 (7.0%)	170 (7.1%)	168 (6.9%)	138 (6.6%)
Afar	57 (2.0%)	50 (2.1%)	49 (2.0%)	42 (2.0%)
Amhara	579 (20.2%)	490 (20.5%)	491 (20.3%)	427 (20.4%)
Oromiya	1259 (43.9%)	1029 (43.0%)	1063 (43.9%)	923 (44.0%)
SNNP	661 (23.1%)	563 (23.5%)	555 (22.9%)	484 (23.1%)
Addis Ababa	111 (3.9%)	90 (3.8%)	93 (3.8%)	81 (3.8%)
Wealth quintile^b^				
Lowest	577 (20.1%)			
Lower	572 (19.9%)			
Middle	572 (19.9%)			
Higher	577 (20.1%)			
Highest	570 (19.8%)			
Education				
Never attended	1181 (41.2%)			
Primary	1152 (40.2%)			
Secondary & higher	535 (18.6%)			
Intended this pregnancy?				
Desired at time	1429 (63.8%)			
Mistimed	598 (26.7%)			
Wanted no more children	211 (9.4%)			
Prefer a home birth?	723 (32.3%)			
Other wives?				
Yes	255 (9.1%)			
No	2546 (90.8%)			
Do not know	2 (0.1%)			
Ever used contraception before this pregnancy?	1747 (60.9%)			
PPFP counseling^c^				
No ANC visit	1539 (53.7%)	621 (26.0%)		
No PNC visit	2713 (94.6%)	1150 (48.1%)		
Counseling at ANC visit	156 (5.5%)	359 (15.0%)		
Counseling at PNC visit	39 (1.4%)	344 (14.4%)		
Counseling at both PNC & ANC visit	15 (0.5%)	134 (5.6%)		
Contraceptive use and intention at each wave				
Does not intend to use	122 (4.3%)	626 (26.2%)	445 (18.4%)	428 (20.4%)
Intends to use	460 (16.0%)	1370 (57.3%)	937 (38.8%)	629 (30.0%)
Unsure of future use	–	–	27 (1.1%)	20 (1.0%)
Using a method	31 (1.1%)	395 (16.5%)	911 (37.7%)	902 (43.1%)
Pregnant	2241 (78.1%)	0 (0.0%)	47 (2.0%)	42 (2.0%)

^a^All estimates are adjusted by wave-specific weights, unless otherwise indicated. Weighted counts are rounded to the nearest whole number. Weighted proportions are rounded to the nearest tenth

^b^Data on wealth quintile, level of education, pregnancy intentions, home birth desires, presence of other wives, and prior contraceptive use were only collected at baseline

^c^Questions about PPFP counseling were only asked at baseline and 6-week follow-up

For our analysis, we merge all four waves of data, so that we follow individual respondents through their baseline and all three follow-ups. The cluster analysis of trajectories necessarily considers only women who are present for all four waves of data.

### Actualization

We define actualization as the behavioral outcome of a respondent’s stated plan. That is, if a woman states that she wants to use contraceptives in the next 12 months, and she is using a method 12 months later, she has actualized her intention to use. At the same time, a woman who states that she does not want to use contraceptives in the next 12 months, and who is not using a method during that period, has actualized her intention to not use. Women who have different outcomes than their stated intentions may have changed intent but also may have faced barriers to their plan, or they may have encountered coercion to change their plan.

Intent-to-use (ITU) contraception is not a new concept in the family planning field, but definitions around this concept have varied widely (see [8] for a review). Unlike the constructed variable *unmet need*, ITU is considered a woman-centered metric, as it asks women directly about their plans, rather than indirectly assessing their need (from the researchers’ perspective). Relatedly, ITU is shown to be more predictive of future use than unmet need is not [34].

Panel data from the PMA that has included measures of intentions and actual contraceptive use have enabled the study of contraceptive actualization. For instance, Magalona et al. [27] outline the link between contraceptive intention and actual use for this Ethiopian cohort, showing that women who sustained an intention to use across multiple time points were more likely to adopt a method by the one-year follow up than those who had not.

### Cluster trajectory analysis

We extend on previous actualization analyses by using cluster analysis on individual postpartum contraceptive trajectories. Cluster analysis, broadly, is an exploratory method that leverages data structure to identify and recognize patterns in data [22]. In this case, cluster analysis allows us to explore individual-level characteristics that make it more or less likely that women will actualize their postpartum contraceptive intentions. When applied to trajectories, cluster analysis will group individual respondents based on the sequence of actions taken from one timepoint to the next. These groups can help us understand major patterns in the pathway from expressing intentions to acting on those intentions and can help us understand the features of respondents who are able to act on their intentions.

We code women’s intent to use alongside their actual use and pregnancy status. Thus, at any given time, women can be pregnant, intend to use, not intend to use, be unsure of future use, or be using a contraceptive method. To measure intent to use, the PMA survey asks the women in the cohort at baseline if they think they will use a contraceptive method to delay or avoid getting pregnant at any time in the future. The survey also asks respondents when they want to use a contraceptive method. We consider this timing while coding the baseline variable—women who want to use a method in the next 12 months are considered to have an intention to use. This allows us to examine how those near-term intentions are actualized (or not) within the corresponding period. While PMA also asks about the timing of future use for the follow up waves, we have not incorporated intention timing for those follow ups, due to a large amount of missing and ambiguous data. For instance, at the baseline, only 226 women specified a year or month of use, while all other respondents offered other time frames, such as “when I’m done breastfeeding” or “when my menses return” or “soon.” Thus, our coding for the follow-up waves is simple: we treat women who answer “yes” as intending to use, women who answer “no” as not intending to use, and women who answer that they “do not know” as unsure of their future use.

Using the R packages ‘cluster’ [26], we conduct a k-means clustering of the full trajectories of the recoded ITU variable as described above. This method of clustering is a straightforward and common approach, which attempts to partition *n* data points into *k* clusters by reducing the distance between data points within each cluster. The method is widely applicable due to its flexibility. It is useful to identify clusters of similar data (in this case, trajectories) which can then be further analyzed.

### Multinomial modeling

In this work, we further analyze the clusters identified in k-means clustering by describing the clusters and estimating a model to understand significant features distinguishing women assigned to each cluster. To do so, we assign each woman in the sample to a cluster based on her observed trajectory. We then estimate a multinomial logistic regression model to examine the multidimensionality of cluster membership. Because the clusters are based only on the contraceptive intent-to-use trajectory, we can examine how other factors, such as socioeconomic status, health-seeking behavior, and demographic characteristics vary across the clusters.

The inclusion of factors associated with health-seeking behavior allows us to further contextualize our analysis by including salient variables like home births and PPFP counseling. High mortality rates in Ethiopia have drawn increased attention to the low utilization of maternal healthcare services. Many women still deliver their babies at home without skilled antenatal care, delivery assistance, or postnatal care [40]. According to the 2016 Ethiopian Demography and Health Survey (EDHS), only 32% of women complete the recommended four antenatal care visits throughout their pregnancy [9]. While these figures have improved over time, they are still low by WHO standards and merit further study.

## Results

The PMA-Ethiopia pregnancy cohort consists of roughly 2,000 women across all four waves. Table 1 presents the descriptive statistics of the sample. At baseline, the respondents ranged from 15 to 47 years old, with a median age of 27. Respondents were distributed across six regions, with approximately 44% of respondents in Oromiya, 23% in SNNP, and 20% in Amhara. Fewer women resided in Afar (2%) and Addis (~ 4%). With survey weights adjusting the raw data, women were evenly distributed over wealth categories. Nearly one in five women had higher than secondary education. Most respondents reported that this pregnancy was desired at the time (64%), with 27% reporting that this pregnancy was mistimed, and only 9.4% reporting that they had wanted no more children before this pregnancy. One in three respondents desired a home birth for this pregnancy. Most respondents (60.9%) had used a contraceptive method before this pregnancy, and nearly three-quarters of respondents stated that they either intended to use a contraceptive method or were already using a method at the 6-week follow up. However, PPFP counseling amongst the sample was very low—with only 30% received counseling on postpartum family planning methods at either a PNC or ANC visit.

**Fig. 1:**
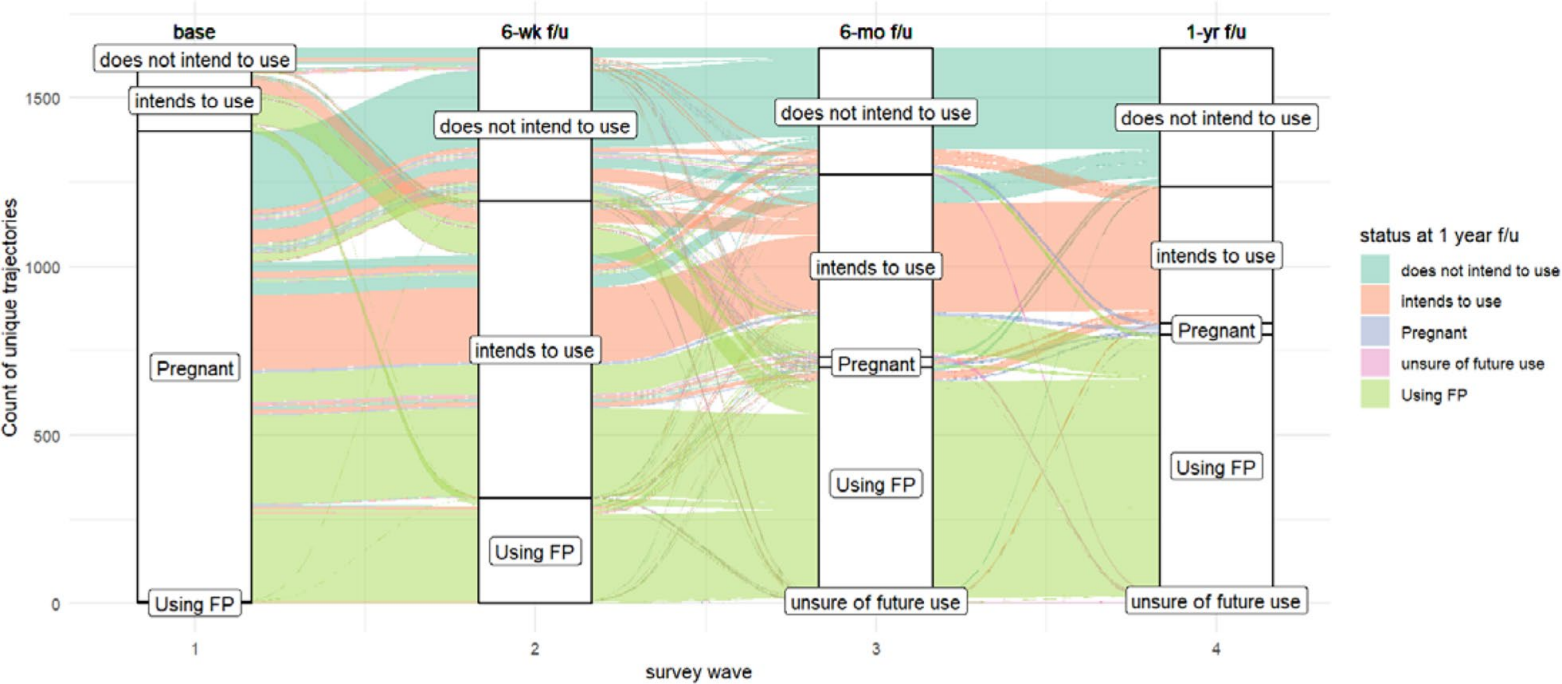
Sankey plot of intent-to-use trajectories for first year postpartum, PMA Ethiopia

Respondents were able to change their reported intentions as well as report actual use or subsequent pregnancies in the 6-week, 6-month, and 1-year follow ups. Figure 1 shows the pathways that the cohort follows, with colors corresponding to their ‘final’ state at the 1-year follow up.

Thus, reading the dark green bar left to right in Fig. 1 shows that the bulk of women who remain ‘non-intenders’ at the 1-year follow up move from pregnancy to non-intention at the 6-week point, but that some move from pregnancy to intention to use at 6-weeks and return to non-intention to use by the end of the 1-year follow up. It also shows a relatively large proportion of women (represented by the salmon bar) who intend to use the entire year postpartum, but never enact that plan.

## Trajectory cluster analysis

Leveraging the panel data structure, we conduct a cluster analysis on the contraceptive intention trajectories that women follow in the first year postpartum. Cluster analyses allow researchers to construct groups based on similar characteristics or patterns within the data [17, 23]. These clusters may be structured hierarchically or non-hierarchically. We use a non-hierarchical method known as k-means clustering [24, 36] because it is well suited for large datasets and it allows individuals to move from one cluster to another, as we depict in Fig. 1. This differs from hierarchical approaches, where an individual cannot move to another cluster once they are assigned.

**Fig. 2:**
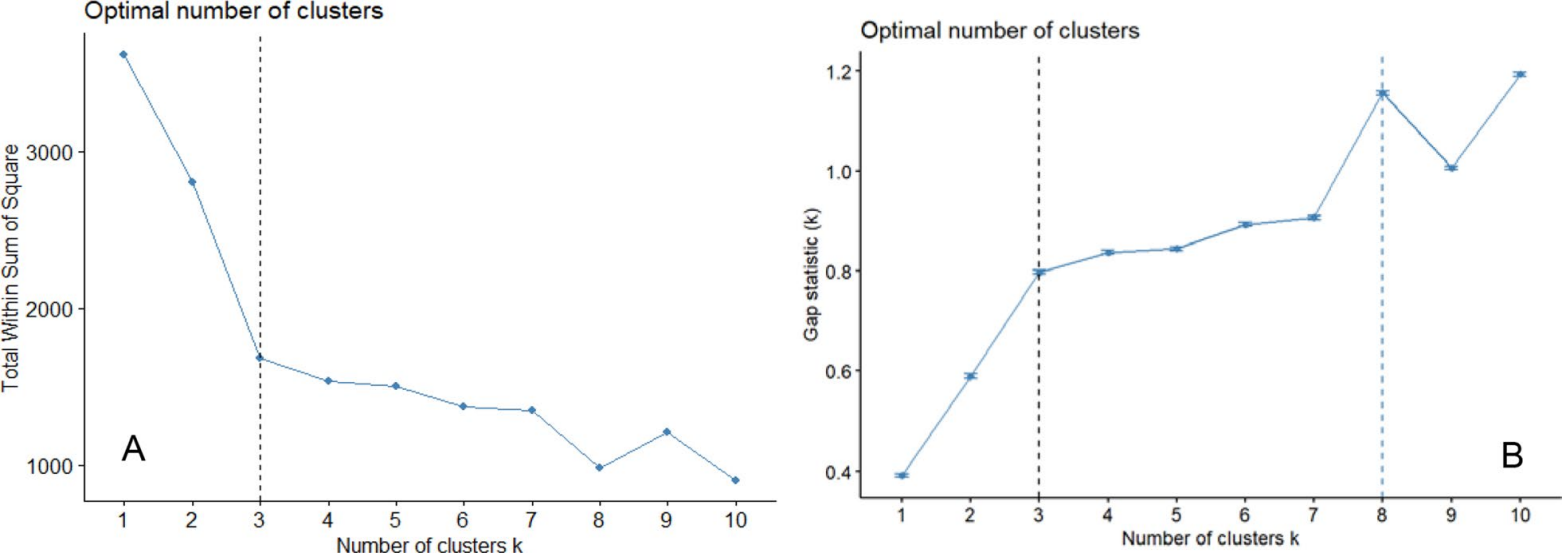
Optimal number of clusters using the elbow method (**A**) and the gap statistic (**B**)

The ‘elbow method’ allows us to visually determine the optimal number of clusters (*k*) for this dataset based on the k-means clustering approach, by finding the inflection point at which the number of clusters introduced produces diminishing returns to the within-cluster sum of squared errors [24]. In the elbow plot in Fig. 2, *k* is increased incrementally and plotted against the total within sum of squares (TWSS). TWSS is a measure of the variability of the observations within each cluster. A cluster that has a small sum of squares is more compact than a cluster that has a large sum of squares. The more compact the cluster, the more similar data points within that cluster are to one another, on the dimension used for clustering.

In Fig. 2, we see a gradual decrease in the within-cluster-sum of squared errors on the x-axis and a sharp inflection point when *k* = 3. This point represents the “elbow” at which we see diminishing returns in the TWSS value as we incrementally add more clusters. As with any cluster analysis, determining the number of clusters makes a difference in the interpretability.

The elbow method works when a strong inflection point emerges. However, we also see in Fig. 2 a dip in TWSS at the k = 8 point. Thus, we supplement the elbow method with the gap statistic method which allows us to compare a heterogeneous, clustered data structure to a hypothetical ‘null’ data structure [47]. The ‘gap’ is the degree to which the real data falls below the expectation of a ‘null’ or normally distributed data structure (i.e. data with no clustering whatsoever). The gap statistic would then identify the number of clusters that create the largest gap between the real and null distributions. We find two inflection points. At k = 8, the gap statistic is maximized, but we also see a similar inflection point at k = 3, reflecting what we found in the elbow method. For the purpose of this analysis, and of interpreting our results, we restrict the algorithm to three clusters. However, we include supplementary materials for a k = 8 clustering algorithm and discuss possible tradeoffs for the two approaches in the limitations section.

**Fig. 3:**
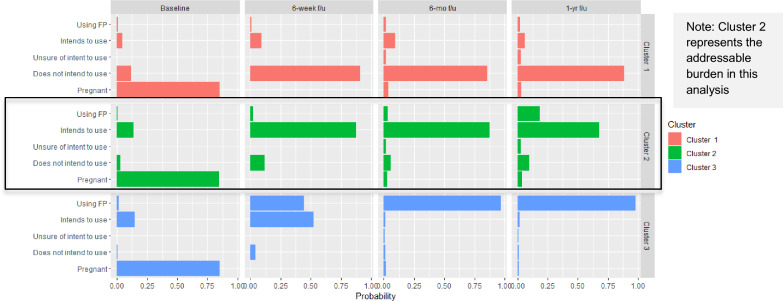
Probabilities of cluster membership based on intent-to-use states across all four waves of the PMA Ethiopia cohort survey

Based on the three-cluster algorithm, we examine which trajectories fall into which clusters. Figure 3, below, shows the probabilities of cluster membership for each state possible for all four waves of the survey. Distinct patterns emerge. Cluster 1 is primarily composed of individuals who do not intend to use a contraceptive method and actualize those intentions to not use during the first year postpartum. For brevity moving forward, we call members of this cluster “Actualized Non-Users.” Cluster 2 is characterized by individuals who intend to use a contraceptive method within the first year, but do not go on to actualize those intentions to use. We label this cluster the “Aspiring Users.” Cluster 3 represents respondents who both express an intent to use PPFP and actualize those intentions. We label this cluster “Actualized Users.”

While the dynamics of Actualized Non-Users and Actualized Users are informative, the cluster of Aspiring Users represents the addressable burden for PPFP in Ethiopia. That is, this is the group of women who have expressed intent to use contraceptives postpartum but who likely face barriers to access, which may be addressed with targeted intervention.

Using the results of the cluster analysis, we assign respondents to the clusters identified based on the probabilities outlined in Fig. 2. Importantly, although the results of the clustering method are probability-based (e.g. what is the probability that a woman who intends to use at 6-weeks and uses at 6-months will be included in Cluster *k*), we use a binary categorization of member or non-member, for the sake of simplifying our interpretation. That is, for a woman whose trajectory matches one of the clusters, she is considered a member, rather than being assigned a value between 0 and 1 to determine how close to a particular cluster she might be. Because of the necessity of categorizing women into mutually exclusive clusters, we allow for women who follow the same trajectory as identified in the clustering algorithm, but who answer in any follow up that they are unsure of their intentions, to be categorized into the cluster, nevertheless. This binary categorization of respondents into clusters necessarily excludes a substantial number of women who do not follow the trajectories identified in the cluster analysis, a limitation that could be more deeply explored with further research. With these simplified clusters, we can examine the individual-level characteristics associated with actualization, as well as with women who were unable to actualize their intent to use. Full descriptive statistics of all three clusters as well as the women who were not assigned a cluster are available in the Supplementary Materials, in table A2. For brevity, we present selected characteristics that differentiate the three clusters in Table 2, below.

**Table 2:**
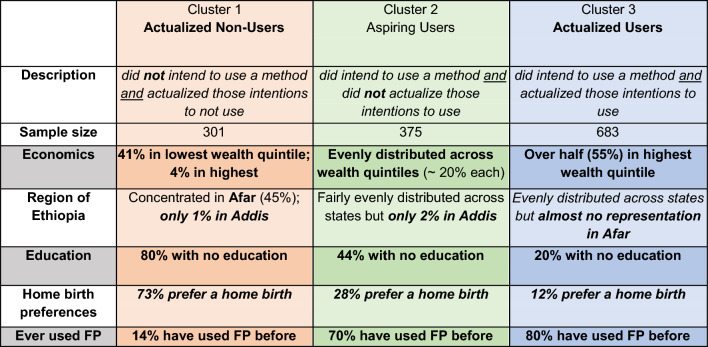
Selected statistics on key individual-level characteristics that differentiate clusters

Examining the individual-level characteristics of respondents in each cluster reveals several distinct patterns. Overall, Aspiring Users were evenly distributed across region and wealth, but 44% had no education (relative to 80% of Actualized Non-Users and 20% of Actualized Users). Contact with the healthcare system is an important factor distinguishing these clusters—while all women were very unlikely to receive PPFP counseling at both ANC and PNC visits, women who expressed intent to use (both Aspiring Users and Actualized Users) were slightly more likely to receive these multiple counseling touchpoints. Over half of women in all clusters did not receive PPFP counseling at any visit. Preference for home birth was highest among Actualized Non-Users (73%), low for Aspiring Users (28%) and lowest for Actualized Users (12%).

**Fig. 4:**
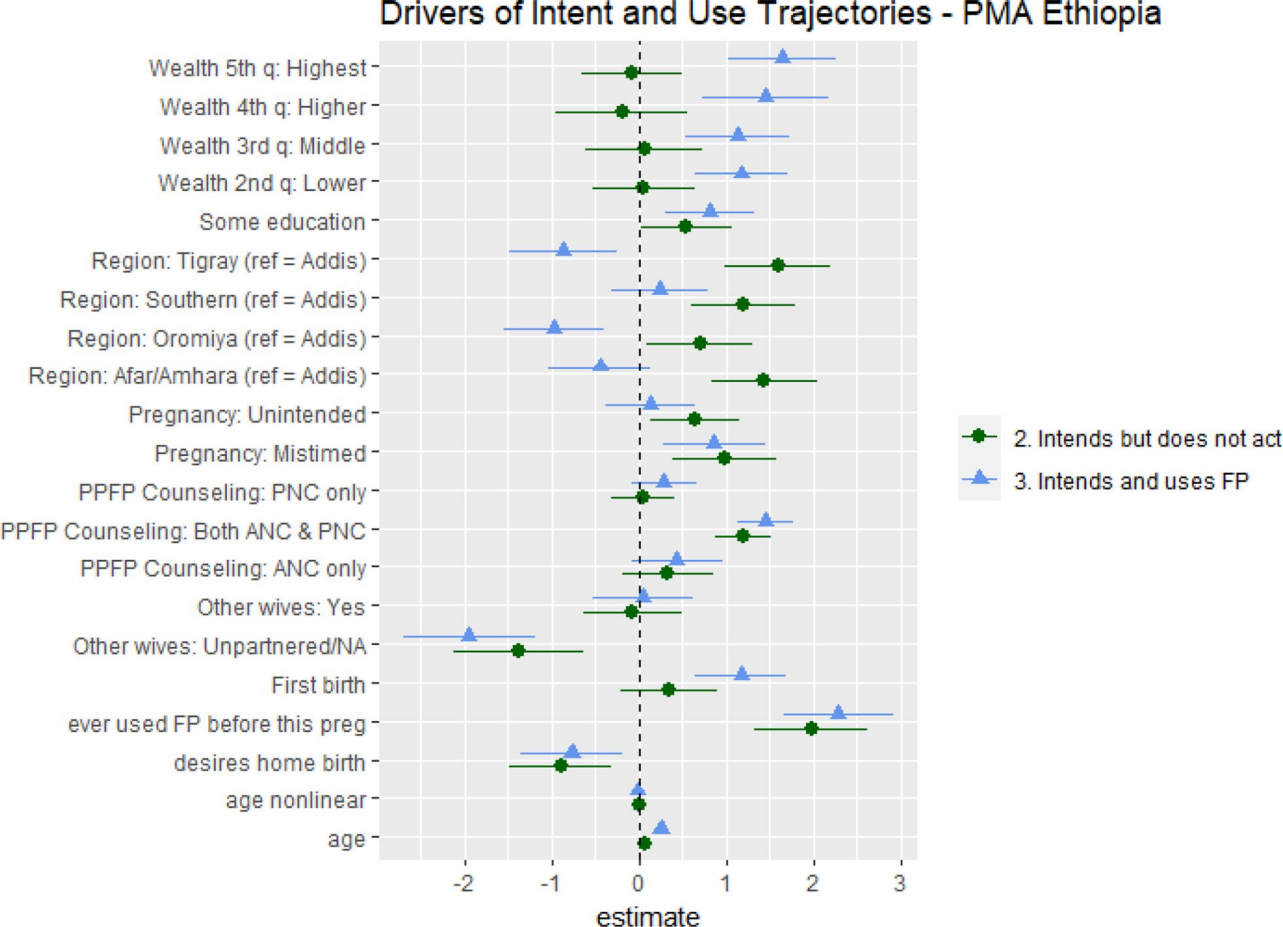
Multinomial regression model coefficients. Reference category is Cluster 1—Actualized non-users

Using the same data and cluster categories, we now turn to the multinomial model results, to estimate the significance of individual factors on a woman’s intent-to-use trajectory. The outcome of the multinomial regression model is cluster membership. Figure 4 below maps coefficients on the x-axis and variables on the y-axis. The vertical dotted line represents the reference, which is the Actualized Non-Users cluster. Point estimates for Aspiring Users (circle) and Actualized Users (triangle) that overlap the dotted line are not significantly different from Actualized Non-Users. Point estimates for Aspiring Users and Actualized Users that overlap is not significantly different from one another. Because we are primarily interested in identifying the drivers of *not* actualizing a stated intent to use, we will focus our main discussion on the variables significantly different for Aspiring Users.

Only two factors were associated with being an Aspiring User rather than actualizing either use or non-use are: (1) this pregnancy being unintended; (2) region. Two additional factors were associated with a significantly higher likelihood of being an Actualized User rather than an Aspiring User or Actualized Non-User: first birth, and wealth. All other factors are either not statistically significant or significant for both Aspiring Users and Actualized Users—suggesting that these factors are significant for developing an intent to use, but not necessarily sufficient for actualizing that intent.

This pregnancy being “mistimed” is associated with higher intentions—with significant higher probability of being an Actualized User or an Aspiring User than an Actualized Non-User, but no difference between intending—as an Aspiring User—and actualizing that intent—as an Actualized User. However, the pregnancy being “unintended” is associated with being an Aspiring User rather than either an Actualized User or an Actualized Non-User. Women who declared their pregnancy truly unintended (i.e., did not want a pregnancy versus wanted a pregnancy but not now) were more likely to intend to use a contraceptive method during the first year postpartum but subsequently not use a method.

Regional differences are striking. Women in Tigray, Oromiya, and the Afar and Amhara regions are more likely to be Aspiring Users, and in the case of Tigray and Oromiya, significantly less likely to be Actualized Users. Perhaps this is not altogether unsurprising, as each of these regions has faced challenges in health care delivery over the same period (e.g., conflict in Tigray).

While we do not see a significant wealth difference between Actualized Non-Users and Aspiring Users, we see a familiar wealth gradient for Actualized Users. This suggests that while wealth does not significantly impact a woman’s intent to use, it certainly seems to play a role in actualizing her intentions. Similarly, if this birth was a woman’s first, she was more likely to be an Actualized User than any other trajectory.

PPFP counseling is a topic that has garnered much attention in Ethiopia, especially with the expansion of the healthcare extension workers nationwide (cite). However, we find that while receiving PPFP counseling in both ANC and PNC visits was significant for women’s expressed intentions, it did not distinguish between women who were Aspiring Users and those who were Actualized Users.

## Discussion

Our analysis reveals who actualizes their contraceptive intentions in the critical first year postpartum. By leveraging panel data and using cluster analysis, we disentangle the individual-level features that enable women to actualize their contraceptive intentions. Wealthier women, women with more educational attainment, and women who had more contact with the formal healthcare system were more likely to actualize their intent to use postpartum family planning. Less wealthy women, women with less education, and women with a higher likelihood of preferring a home birth, were more likely to express no intention to use a contraceptive method, and to remain unchanged throughout the first year postpartum. Although we find these trajectories to be informative, especially in understanding the relationship between contraception and contact with the healthcare system, it is the cluster of women who express intention to use contraception *and who do not actualize those intentions (Aspiring Users)* who represent the addressable burden for Ethiopia’s PPFP intervention programs. Aspiring Users are much more likely to be concentrated outside of Addis Ababa, and more likely to report that this pregnancy was unintended than any other cluster.

Our analysis highlights two missed opportunities for Ethiopia’s maternal health strategy. First, counseling on PPFP options represents a clear gap when it comes to women’s *expressed intention to use*. Few women received counseling at more than one type of visit, but those who did were much more likely to intend to use. However, to complicate this result, we do not see the experience of PPFP counseling distinguishing between women who expressed intent to use and actualized, versus those who did not actualize. This finding suggests that while dedicated PPFP counseling may help women make an informed choice, intrinsically, it does not seem to help her leverage resources to actualize her intention to use contraception. One explanation for this may be related to the quality of counseling in Ethiopia, which has declined precipitously over the same time period, from 39% of women reporting they received high quality counseling in 2015 to only 12% in 2019 [15]. Dedicated investment in *the quality of* PPFP counseling may see an increased impact on actualization.

Second, we find that women in this cohort with an unintended pregnancy are more likely to fall into the Aspiring Users cluster—those who intend to use contraception but do not actualize. This is an interesting expansion of findings from Zimmerman et al. [48], who found that women who reported the pregnancy as mistimed were more likely to adopt contraception in the first year postpartum than those with an unintended pregnancy. We contend that these two findings highlight a linked challenge for women with unintended pregnancies—both that they are less likely to use contraception after an unintended pregnancy AND that even when they intend to use contraception, they are less likely to actualize those intentions. Programs that support women experiencing unintended pregnancies should include dedicated attention to postpartum family planning.

### Limitations

As with all modeling efforts, ours has limitations. We chose a straightforward way of implementing our clusters into our dataset, which excluded a substantial number of individuals from the analysis. Our initial sample included 2,094 women at the 1-year postpartum follow up while our cluster analysis included 1,359 women, resulting in the exclusion of 735 women. Although upon comparison of the three clusters to the excluded women, we do not find systematic biases across the variables of interest, future work could investigate the excluded respondents in more depth. Relatedly, we chose three clusters based on the ‘elbow’ method. Other alternatives are available, and we present one alternative using a k = 8 clustering algorithm in the supplementary materials. These alternative criteria may change the way clusters show patterns, including more information from women who change their intentions during the first year postpartum. Examining those changes could generate further insights into not only who actualizes their contraceptive intentions, but just how dynamic those intentions can be, and who might be more likely to change their minds during the first year postpartum.

## Conclusion

With increased attention to meeting women’s actual expressed preferences and intentions around contraceptive use, it is critical that we understand not just when, but also *how* and *why* women are not able to actualize their family planning intentions. This research attempts to interrogate the conditions under which women in a pregnancy cohort are able to actualize their postpartum family planning intentions. With this specific cohort, we can hold life course events such as pregnancy at a constant and examine this critical time period for contraception—in which rapid repeat pregnancies can have the most devastating mental and physical health consequences. Leveraging data from this pregnancy cohort, we examine clusters of trajectories that women follow from pregnancy to contraceptive intentions to contraceptive behavior. Using cluster analysis we find three distinct trajectories—Actualized Non-Users, Aspiring Users, and Actualized Users. We estimate multinomial regression models to examine the drivers of following one of these three paths.

Our findings indicate that, relative to the Actualized Users and Non-Users, Aspiring Users of postpartum contraception were more likely to report the pregnancy as unintended and were more likely to be concentrated in Tigray and Oromiya. We find that wealth did not differentiate those with no intention and our Aspiring Users but did impact the likelihood of becoming an Actualized User. Finally, we find that PPFP counseling in ANC and PNC visits did not differentiate our Aspiring Users from Actualized Users. Taken together, these findings suggest that structural barriers to actualization likely exist at the regional level, and that women who experience unintended pregnancies are especially susceptible to remain Aspiring Users postpartum.

Future data collection and research are needed to continue to examine the drivers of aligning contraceptive intentions and behaviors. For instance, we suspect that wealth is an enabler of *both* intention to use and subsequent actualization—that is, women who have the economic means to access and use contraceptive methods may also be more likely to see the use of contraceptives as a possible outcome and report an intention to use.

Although we find no significant difference between Aspiring Users and Actualized Users in terms of counseling, we suspect that the quality of such counseling matters. Receiving dedicated FP counseling is only one component of contraceptive autonomy—the quality and personalization of that counseling are important aspects that could be improved in Ethiopia to support women’s ability to actualize their contraceptive intentions.

## Supplementary Information

Supplementary material 1.

Supplementary material 2.

## Data Availability

Open source survey data were obtained with permission from Performance Monitoring in Action and can be accessed at www.pmadata.org.

## Footnotes

1 Due to ongoing conflict in Tigray, women surveyed in this region were subject to higher-than-average loss to follow up. In our analyses, we use wave-specific survey weights that help address this loss to follow up. Nevertheless, readers should interpret Tigray regional dynamics with caution.
