# Supplementary material for: Who actualizes postpartum contraceptive intentions? A trajectory cluster analysis
Source: Reprod Health. 2024 Nov 21;21:169. doi: 10.1186/s12978-024-01899-7 (PMC11583453; doi:10.1186/s12978-024-01899-7)
Supplement: Supplementary file 1 — Supplementary material 1. [file 12978_2024_1899_MOESM1_ESM.pdf]

Table A1. Results of alternative k-means clustering with k=8 clusters

	baseline				6-week follow up			6-month follow up					1-year follow up				
cluster	does not intend to use	intends to use	Pregnant	Using FP	does not intend to use	intends to use	Using FP	does not intend to use	intends to use	Pregnant	unsure of future use	Using FP	does not intend to use	intends to use	Pregnant	unsure of future use	Using FP
1	0	0	1	0	0.054973822	0.945026178	0	0.015707	0.22513089	0.005236	0.002618	0.751308901	0.013089	0	0	0	0.986910995
2	0.042373	0.067797	0.889830508	0	0.983050847	0	0.016949	0.118644	0.813559322	0.016949	0.025424	0.025423729	0.288136	0.59322	0.025424	0.025424	0.06779661
3	0.777778	0.206349	0	0.015873	0.825396825	0.174603175	0	0.825397	0.079365079	0.031746	0.047619	0.015873016	0.809524	0.126984	0.015873	0.031746	0.015873016
4	0	0	1	0	0.111111111	0.888888889	0	0.138889	0.222222222	0.416667	0.111111	0.111111111	0.083333	0.194444	0.638889	0.083333	0
5	0.010929	0.155738	0.833333333	0	0	0.978142077	0.021858	0.060109	0.890710383	0.002732	0.008197	0.038251366	0.120219	0.860656	0	0.019126	0
6	0	0	1	0	0.896193772	0.096885813	0.00692	0.958478	0	0.024221	0	0.017301038	0.941176	0	0.020761	0.020761	0.017301038
7	0	0.069767	0.913621262	0.016611	0	0	1	0.003322	0.023255814	0.013289	0	0.96013289	0	0.016611	0	0	0.983388704
8	0.01087	0.98913	0	0	0.010869565	0.989130435	0	0	0.108695652	0	0.01087	0.880434783	0.01087	0.021739	0.01087	0	0.956521739
