# Supplementary material for: Who actualizes postpartum contraceptive intentions? A trajectory cluster analysis
Source: Reprod Health. 2024 Nov 21;21:169. doi: 10.1186/s12978-024-01899-7 (PMC11583453; doi:10.1186/s12978-024-01899-7)
Supplement: Supplementary file 2 — Supplementary material 2. [file 12978_2024_1899_MOESM2_ESM.pdf]

Table A2. Unweighted descriptive statistics based on cluster[1]				
	Cluster 1	Cluster 2	Cluster 3	Not assigned
Sample size	301	375	683	1057
Age				
Range	15-45	15-46	15-47	15-45
25th percentile	23	24	22	22
Median	28	28	26	26
75th percentile	33	33	30	30
Regional distribution				
Tigray	23 (7.6%)	91 (24.3%)	117 (17.1%)	179 (16.9%)
Afar	135 (44.9%)	5 (1.3%)	3 (0.4%)	69 (6.5%)
Amhara	21 (7.0%)	84 (22.4%)	123 (18.0%)	185 (17.5%)
Oromiya	62 (20.6%)	102 (27.2%)	153 (22.4%)	270 (25.5%)
SNNP	57 (18.9%)	85 (22.7%)	158 (23.1%)	261 (24.7%)
Addis Ababa	3 (1.0%)	8 (2.1%)	129 (18.9%)	93 (8.8%)
Wealth Quintile				
Lowest	122 (40.5%)	77 (20.5%)	43 (6.3%)	174 (16.5%)
Lower	48 (15.9%)	70 (18.7%)	58 (8.5%)	180 (17.0%)
Middle	58 (19.3%)	76 (20.3%)	94 (13.8%)	150 (14.2%)
Higher	58 (19.3%)	67 (17.9%)	114 (16.7%)	224 (21.2%)
Highest	15 (5.0%)	85 (22.7%)	374 (54.8%)	329 (31.1%)
Education				
Never attended	236 (78.4%)	164 (43.7%)	142 (20.8%)	378 (35.8%)
Primary	50 (16.6%)	146 (38.9%)	271 (49.7%)	392 (37.1%)
Secondary & higher	15 (5.0%)	65 (17.3%)	270 (39.5%)	287 (27.2%)
Intended this pregnancy?				
Desired at time	216	197	494	542

	(71.8%)	(52.5%)	(72.3%)	(51.3%)
Mistimed	39 (13.0%)	89 (23.7%)	156 (22.8%)	185 (17.5%)
Wanted no more children	14 (4.7%)	39 (10.4%)	28 (4.1%)	74 (7.0%)
N/A	32 (10.6%)	50 (13.3%)	5 (0.7%)	256 (24.2%)
Prefer a home birth?	190 (63.1%)	86 (22.9%)	79 (11.6%)	223 (21.1%)
Other wives?				
Yes	58 (19.3%)	26 (6.9%)	34 (5.0%)	90 (8.5%)
No	237 (78.7%)	338 (90.1%)	643 (94.1%)	920 (87.0%)
Do not know	1 (0.3%)	3 (0.8%)	0 (0%)	1 (0.1%)
Ever used contraception before this pregnancy?	42 (14.0%)	261 (69.6%)	547 (80.1%)	616 (58.3%)
PPFP counseling				
no PPFP counseling	35 (11.6%)	114 (30.4%)	303 (44.4%)	277 (26.2%)
counseling at ANC visit	6 (2.0%)	26 (6.9%)	52 (7.6%)	57 (5.4%)
counseling at PNC visit	5 (1.7%)	23 (6.1%)	85 (12.4%)	80 (7.6%)
counseling at both PNC & ANC visit	5 (1.7%)	24 (6.4%)	70 (10.2%)	63 (6.0%)
N/A – No visit or refused	250 (83.1%)	188 (50.1%)	173 (25.3%)	580 (54.9%)

Notes:
[1]Percentages are calculated by column to facilitate comparison across clusters.
